# Prevalence of gastrointestinal parasites in cattle and sheep in three municipalities in the Colombian Northeastern Mountain

**DOI:** 10.14202/vetworld.2019.48-54

**Published:** 2019-01-08

**Authors:** Juan Carlos Pinilla León, Nelson Uribe Delgado, Angel Alberto Florez

**Affiliations:** 1 Department of Veterinary Medicine, University of Santander, Faculty of Exact, Natural and Agricultural Sciences, Animal Science Research Group, Bucaramanga, Colombia; 2 Department of Parasitology, Industrial University of Santander, Faculty of Health, Research Group in Molecular Epidemiology, Bucaramanga, Colombia

**Keywords:** cattle, gastrointestinal parasites, prevalence, sheep

## Abstract

**Aim:**

The study was conducted to determine the prevalence of gastrointestinal (GI) parasites in cattle and sheep from three municipalities in the Colombian Northeastern Mountain.

**Materials and Methods:**

Overall, 200 fecal samples were collected directly from the rectum in cattle and sheep. The presence of helminths eggs and coccidial oocysts in fecal samples was detected using McMaster and Dennis techniques. Identification of eggs or oocysts was done on the basis of morphology and size of the eggs or oocysts.

**Results:**

The global prevalence of GI parasites was 56.3%. Regarding the prevalence by municipalities, there was no statistical association (p>0.05), indicating that the prevalence was similar in the three municipalities. The prevalence of parasitic infection was higher in sheep (63%) as compared to that of cattle (50.5%), but the difference was nonsignificant (p>0.05). The most prevalent parasites were *Eimeria* spp., *Fasciola hepatica*, and Strongylida order. Regarding the results for *Eimeria* spp., different degrees of positivity were observed, but there was no statistical association (p>0.05) with respect to the age group. Likewise, there was no statistical association (p>0.05) between the prevalence for Strongylida order and *F. hepatica* with respect to the age group.

**Conclusion:**

Cattle and sheep in Colombian Northeastern Mountain were infected with helminths and coccidia. The prevalence values of GI parasites were moderate in both species warranting treatment. The presence of *F. hepatica* represents a risk factor to health public. Future studies are required to evaluate the parasitic dynamics throughout the year and the impact on animal production.

## Introduction

Gastrointestinal (GI) parasitism is a disease caused by different genera of parasites that inhabit the digestive tract of cattle and sheep, causing inappetence, anemia, diarrhea, poor growth, and economic losses in the herds. Basically, GI parasitism in cattle and sheep is caused by helminths and protozoa [1]. *Eimeria* spp. is a protozoa belonging to the phylum Apicomplexa, family Eimeriidae that parasitize poultry, ruminants, equines, and rabbits, which causes bovine and ovine coccidiosis [1]. Helminthes are parasites that cause parasitic gastroenteritis in cattle and sheep. Among the nematodes, the most important and prevalent genera worldwide are those belonging to the Strongylida order, especially in tropical zones [2]. *Fasciola hepatica* is a trematode parasite affecting cattle, sheep, and occasionally a man, which requires an intermediate host for their transmission. In Colombia, the national prevalence for *F. hepatica* in bovines is 25%, with ranges between 25% and 80% in the Boyacá, Nariño, and Cundinamarca states [3]. *Paramphistomum* spp., *Cotylophoron* spp., and *Calicophoron* spp. are paramfistomids of veterinary importance, responsible for paramfistomosis, diagnosed in bovines, buffaloes, camelids, goats, and sheep [4].

The municipalities of Encino, Duitama, and Belen are located in the Northeastern Colombian Mountain. It is an agricultural region with traditional and small-scale livestock farms, being the cattle and sheep husbandry one of the most important components of the local economy. This region is notorious for its small dairy industry; however, 90% of the farms are dedicated to dual purpose (beef and milk). According to ICA’s vaccination records, the animal population census for the three municipalities was 15,000 heads [5].

In Colombia, there is very little epidemiological information on GI parasitism in cattle and sheep, mainly in the Eastern Mountain of the country, and for this reason, the aim was to determine the prevalence of GI parasites in cattle and sheep in the municipalities of Encino, Santander state, and Duitama and Belen, Boyaca state, Colombia.

## Materials and Methods

### Ethical approval

This research was approved by the Institutional Ethical Committee of the University of Santander and Industrial University of Santander, Colombia.

### Study area

**Figure-1:**
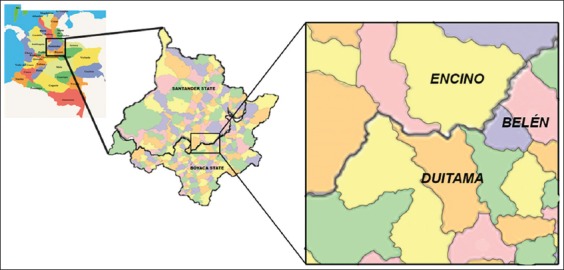
The political map of Colombia (left) and the study area (middle and right) with the three municipalities (Encino, Duitama, and Belen).

The study was conducted in the municipalities of Encino (6°08′16″N-73°05′53″O) in the state of Santander and Duitama municipality (5°49′19″N-73°01′47″O) and Belen municipality (5°59′22″N-72°54′44″O) in the state of Boyaca, Colombia (Figure-1). Bioclimatic characteristics of the region are a mean annual temperature of 15°C, with little weather variation along the year. Altitude is between 1850 and 4200 msl and mean annual rainfall is 1815 mm, with 87% of relative humidity [6,7].

### Study design and sampling

A random sampling, descriptive and transversal, was designed. 34 traditional and small-scale livestock farms were visited between October 2017 and February 2018, with a predominance of dairy breeds. The grazing management system and concentrated foods were followed in the animals. The average of animals per farm ranges from 30 to 35. Using the formula for known populations [8], with an expected prevalence of 25% [9], and a confidence level of 95% with 6% of associated maximum error, 200 fecal samples were determined. 6-8 fecal samples were collected from each farm examined. Animals were categorized according to the age in: <12 months, 12-24 months, and >24 months.

### Collection and examination of fecal samples

The study was conducted on 200 animals (103 cattle and 97 sheep) of small dairy farm. The fecal samples were collected randomly. Approximately 5-10 g of feces were collected directly from the rectum from each bovine and sheep, using previously labeled polyethylene bags. Samples were refrigerated and processed in the Investigation Research Laboratory of the Parasitology of the Industrial University of Santander. The stool samples were cultivated at room temperature in Petri dishes using 20 mL of 2.5% potassium dichromate solution for 24 h and later processed by the McMaster technique to determine the oocysts per gram (OPG) of feces and eggs per gram (EPG) of feces. The numbers of OPG and EPG were calculated with a detection level of 50 (two chambers) which is the standardized factor for this technique [10]. The Modified Dennis technique [11] was employed to detect the heavy *F. hepatica* and *Paramphistomum* spp. eggs. The oocysts and eggs of parasites were identified from their morphological characters, using a light optical microscope with a magnification of 10× and 40×.

### Statistical analysis

The GI parasitism prevalence results were analyzed by descriptive statistics and the Chi-square test to determine the analyzed variables. Calculations were made using the SPSS version 21 [12].

## Results

The overall prevalence of GI parasites in the three municipalities was 56.5% (113/200). No statistical association was found (χ^2^=4.9; p>0.05) between prevalence values in the three municipalities: 54.5% (42/77) in Encino, 59% (59/100) in Belen, and 52.1% (12/23) in Duitama. According to these results, the prevalence is present in similar proportions in the three municipalities of the Santander and Boyaca state, Colombia. Regarding to results in cattle and sheep, the prevalence of GI parasites in cattle was 50.4% (52/103) and 62.9% (61/97) in sheep. No statistical association was found (χ^2^=2.7; p>0.05) between prevalence values in the two examined species. According to these results, the prevalence is present in similar proportions in cattle and sheep. With respect to the prevalence of GI parasites and sex of the animals, different degrees of parasitism were observed in female and male, but no statistical association was found (p>0.05) with respect to the sex.

Table-1 shows the seven parasite genera found in the study, being *F. hepatica* (22.3%), *Eimeria* spp. (17.4%), and Strongylida order (16.5%) the most prevalent in cattle, while in sheep, the most prevalent values were found in Strongylida order (31.9%) followed by *Eimeria* spp. (30.9%) and *F. hepatica* (14.4%). As for the intensity of infection, *Eimeria* spp. showed the highest level (820 and 1270 OPG) in cattle and sheep, respectively, followed by Strongylida order (150 and 385 EPG) in cattle and sheep, respectively. *F. hepatica*, *Paramphistomu* m spp., and *Moniezi* a spp., were not counted. The genera of nematodes and trematodes were identified by the morphology and size of their eggs [13].

**Table-1:** Prevalence and intensity of infection of GI parasites in cattle and sheep from the three municipalities.

Parasite	Cattle (n=103)		Sheep (n=97)	
	
	Positive (%)	Intensity of infection (EPG-OPG)	Positive (%)	Intensity of infection (EPG-OPG)
*Eimeria* spp.	18 (17.4)	820	30 (30.9)	1270
Strongylida*	17 (16.5)	150	31 (31.9)	385
*Fasciola hepatica*	23 (22.3)	-	14 (14.4)	-
*Paramphistomum* spp.	1 (0.97)	-	2 (2.06)	-
*Strongyloides* spp.	4 (3.8)	110	3 (3.1)	333
*Moniezia* spp.	1 (0.97)	-	7 (7.21)	-
*Trichuris* spp.	0 (0)	0	2 (2.06)	75

* Reported as order; *Fasciola hepatica, Paramphistomum* spp., *Moniezia* spp. were not counted, EPG=Eggs per gram of feces, OPG=Oocysts per gram of feces

Table-2 shows the comparison (Chi-square test) between positive percentages of *Eimeria* spp. parasitism and age of the animals. Different degrees of parasitism were observed in cattle and sheep, but no statistical association was found (p>0.05) with respect to the age group. Although all group showed infection by coccidian infection, cattle and sheep >24 months showed lower prevalence (15.3% and 40%), respectively, than those under 12 months (23.5% and 48.5%).

**Table-2:** Comparison between prevalence of *Eimeria* spp. infection and age group in cattle and sheep.

Age group	Cattle	Sheep
	
	Positive (%)	Positive (%)
<12 months	4 (23.5)	16 (48.5)
12-24 months	3 (50)	8 (24.2)
>24 months	11 (15.3)	6 (40)
Total	18 (17.4)	30 (30.9)
Chi-square	χ^2^=4.6; p=0.09	χ^2^=4.2; p=0.1

No statistically significant (p>0.05)

Table-3 shows the comparison (Chi-square test) between positive percentages of trematodes and cestodes parasitism and age of the animals. Different degrees of parasitism were observed in cattle and sheep, but no statistical association was found (p>0.05) with respect to the age group. All group showed infection by *F. hepatica* in cattle, but all the animals <24 months did not evidence excretion of *Paramphistomum* spp. eggs, while cattle >12 months did not evidence excretion of *Moniezia* spp. eggs. Sheep did not show excretion of *Paramphistomum* spp. eggs in <12 months animals. Table-4 shows prevalence values for the nematodes observed during the study. The parasites of the Strongylida order presented the highest prevalence in cattle and sheep (17.9% and 38.3%), respectively, followed by the genus Strongyloides (4.2% and 3.7%), respectively. *Trichuris* spp. eggs were not found in fecal samples of cattle, while in sheep, two animals (2.4%) <12 months showed infection by this parasite. The comparison (Chi-square test) between positive nematode values and age group did not revealed statistical (p>0.05) association between prevalence of parasite of the Strongylida order, *Strongyloides* spp., and *Trichuris* spp. and the age of the animals (Table-4).

**Table-3:** Comparison between the prevalence of Trematoda and Cestoda parasitism and age group in cattle and sheep.

Age group	*Fasciola hepatica* Positive (%)	*Paramphistomum* spp. Positive (%)	*Moniezia* spp. Positive (%)
Cattle (n=103)			
<12 months	2 (11.7)	0 (0)	1 (5.8)
12-24 months	1 (16.6)	0 (0)	0 (0)
>24 months	20 (27.7)	1 (1.4)	0 (0)
Total	23 (22.3)	1 (0.97)	1 (0.97)
Chi-square	χ^2^=2.3; p=0.3	χ^2^=0.3; p=0.8	χ^2^=4.6; p=0.9
Sheep (n=97)			
<12 months	5 (15.1)	0 (0)	4 (12.1)
12-24 months	8 (24.2)	1 (3)	1 (3)
>24 months	1 (6.6)	1 (6.6)	2 (13.3)
Total	14 (14.4)	2 (2.06)	7 (7.2)
Chi-square	χ^2^=2.4; p=0.3	χ^2^=1.9; p=0.3	χ^2^=2.2; p=0.3

No statistically significant (p>0.05)

**Table-4:** Comparison between prevalence of Nematoda parasitism and age group in cattle and sheep.

Age group	Strongylida* Positive (%)	*Strongyloides* spp. Positive (%)	*Trichuris* spp. Positive (%)
Cattle (n=103)			
<12 months	2 (11.8)	1 (5.9)	0 (0)
12-24 months	2 (33.3)	0 (0)	0 (0)
>24 months	13 (18.1)	3 (4.2)	0 (0)
Total	17 (16.5)	4 (3.8)	0 (0)
Chi-square	χ^2^=1.4; p=0.5	χ^2^=0.38; p=0.8	χ^2^=0; p=0
Sheep (n=97)			

**Age group**	Strongylida* Positive (%)	***Strongyloides* spp. Positive (%)**	***Trichuris* spp. Positive (%)**

<12 months	12 (36.4)	1 (3)	2 (6.1)
12-24 months	16 (48.5)	1 (3)	0 (0)
>24 months	3 (20)	1 (6.7)	0 (0)
Total	31 (31.9)	3 (3.1)	2 (2.06)
Chi-square	χ^2^=3.6; p=0.1	χ^2^=0.45; p=0.8	χ^2^=2.9; p=0.22

* Reported as order; no statistically significant (p>0.05)

## Discussion

The GI parasitism is one of the major health problems affecting the productivity of the cattle and sheep in worldwide [14]. The presence of GI parasites in cattle and sheep depends greatly on predisposing environmental factors such as temperature and humidity. This has been observed in some parasites of the Strongylida order, which prevail in cold climates or tropical conditions [15]. The general prevalence (56.3%) of GI parasites agrees with the findings of Colina *et al*. [16], who reported high prevalence in cattle in different regions of Peru with climatic conditions similar to those of Encino, Duitama, and Belen municipalities. Possibly, the environmental characteristics, reproductive stage, sex of the animal, as well as the pasture and agricultural practices in the farms have been predisposing factors for the high parasite prevalence in the cattle and sheep under study. These factors play a determinant role in the presence of the infective stages, favoring the developing of the reproductive cycles and the viability of eggs and larvae, which, in turn, depend on the season of the year, the age, and the immune status of the host [16].

The prevalence found in the three municipalities was similar, since the temperatures and humidity conditions in the zone, the type of vegetation, the management in most of the farms, sanitary programs, and management of pastures in the control of infectious agents are very much the same in the three municipalities.

The GI parasites in cattle and sheep are considered one of the most important in tropical herds, since they reduce weight gain and cause high morbidity and mortality in young animals [1,17]. The present study found that *F. hepatica* was most prevalent parasite in cattle (22.3%) followed by *Eimeria* spp. (17.4%) and parasite genera grouped under the Strongylida order (16.5%), while in sheep, the Strongylida order was most prevalent (31.9%). These results agree with those by Orjuela *et al*. [18] who reported 26.8% of coccidian infection in cattle of the North Coast of Colombia. However, these results differ from those reported by Pinilla *et al*. [19] who found high prevalence (77.9%) of *Eimeria* spp. in cattle of Aguachica and Rio de Oro municipalities, Cesar state, while Diaz de Ramirez *et al*. [17] reported 53% prevalence in cattle from Trujillo state, Venezuela, and 86.01% prevalence in cattle from Yucatan state, Mexico [20,21]. Regarding to prevalence by *Eimeria* spp. in sheep, the result differs with those by Ensuncho-Hoyos *et al*. [22], Díaz-Anaya *et al*. [23], Pulido-Medellín *et al*. [24], who reported prevalence 81.6%, 94.4%, and 63%, respectively, in Cordoba and Boyaca state, Colombia. The mean intensity of infection for *Eimeria* spp. found in this study (820 OPG and 1270 OPG) in cattle and sheep, respectively, is considered moderate [15], it is inferred that could be caused by immunosuppression in the animals due to factors such as stress associated with overpopulation, transportation, and herd movements [25]. The coccidia infection in bovine and sheep could be due to their ability to adapt to different climatic conditions as well as for pasture management and contamination of water by parasitized adult cattle [26]; however, the low prevalence found in this study could be associated with the absence of risk factors, such as time of the year (sampling time), low rainfall in the area, size of the farm (200-300 bovines), and period (rainy season) [15].

Regarding to *Eimeria* spp., prevalence and age of the animals indicated that the infection in cattle and sheep occurs in any period during life and with higher excretion of oocysts in young animals (under than 24 months). The results obtained in cattle agree from those reported by Díaz de Ramírez *et al*. [17] and Tomczuk *et al*. [27] who found higher excretion of oocysts in young animals, while the infection in adult decreases. This is attributed to a considerable percentage of calves excreting oocysts during their 1^st^ month of life, and as most bovine Eimeria species have prepatent periods ranging from 2 to 3 weeks, calves most ingest a sufficient amount of sporulated oocysts to establish a patent infection in the herd. In the present study, infected adult cattle and sheep (15.3% and 40%), respectively, could become as asymptomatic host and potential sources of infection for calves and lambs, as they become infected when inoculated with sporulated oocysts by water, or licking the hair of animals with contaminated feces [28].

The prevalence values found for *F. hepatica* in cattle (22.3%) and sheep (14.4%) agree from those reported by other studies conducted in Colombia, reporting a national prevalence of 25%, using coprological techniques [3]. Furthermore, the results obtained agree from those reported by Soca-Pérez *et al*. [29], Ticona *et al*. [30], and Gauta *et al*. [31], who reported medium and high prevalence values in farms from Cuba, Peru, and Venezuela, respectively. However, the results obtained in this study differ with those reported by other authors, who found lower prevalence values in cattle from different Costa Rica regions, Quindio and Cesar state, Colombia, respectively [32,33]. Furthermore, the result differs with those reported by Pulido-Medellin *et al*. [24], who determined lower prevalence values in sheep from Toca, Boyaca. Although there are no previous studies on prevalence and condemnation of livers from animals coming from the study zone, the medium prevalence values for *F. hepatica* found could be due to the interaction between susceptible animals and intermediate hosts who require special climatic conditions for their survival [29]. Moreover, the present study was designed as transversal and conducted in a dry season; this means that the dynamics of the parasite in other seasons of the year is not known.

According to the classification indicated by Valderrama [34] to define the *F. hepatica* endemicity area, the municipalities under study are considered as mesoendemic zones (10-50% prevalence). This region is located at high altitudes (1800-3500 masl), with low temperatures (13-18°C), and mean precipitation rates between 1130 mm and 2500 mm, which are favorable climatological characteristics for the presence of intermediate hosts. Therefore, breeding in grass favors the presence of the parasite, since the animals have direct contact with the infecting form [34]. The ideal temperature for the trematode cycle should be between 10°C and 30°C, with the presence of rain for 3 or more months a year. However, the temperature and humidity determine the seasonality of the disease [15]. Therefore, the characteristics of the region studied are highly favorable for the presence of limneid snails and the development of *F. hepatica*. Therefore, the control of this parasitism is very important, not only for its economic impact on cattle and sheep husbandry but also for presenting a public health problem due to its condition of an emerging zoonosis.

*Paramphistomum* spp. showed prevalence 0.97% and 2.06% in cattle and sheep, respectively. *Paramphistomum* spp., *Cotylophoron* spp., and *Calicophoron* spp. are paramfistomids of veterinary importance, responsible for paramfistomosis, diagnosed in cattle, buffaloes, camelids, goats, and sheep [4]. In Colombia, *Paramphistomum* spp. and *Cotylophorum* spp. have been reported in bovine from the Caribbean Coast, as well as in farms from Antioquia [35], Cundinamarca, Casanare, and Meta state [36,37], as well as in sheep from Central Andean region [38]. Therefore, this result is very important since this parasitic genus has not been reported in cattle and sheep of the municipalities under study and could serve to search other studies for this parasitosis. The similarity in morphologic characteristics of paramphistomidae species as well as the lack of expert trematologists becomes an obstacle for epidemiological prevalence studies. Therefore, the employment of genomic sequences is recommended [39]. It is inferred that the low prevalence for this trematode in Encino, Duitama, and Belen municipalities could be associated with the low occurrence of the specific intermediate molluscs hosts for this trematode (Lymnaea genus snails); however, it could be thought that a biological competition between the *F. hepatica* and *Paramphistomum* spp. miracidiae by parasitizing intermediate molluscs is the reason for the low prevalence [39].

As for the cestodes group, *Moniezia* spp. eggs in cattle were very low (0.97%) and no differences were found between the three age groups. This result agrees with those reported by Orjuela *et al*. [18] and Rodríguez-Vivas *et al*. [21], who demonstrated similar prevalence values. Unlike cattle, the presence of *Moniezia* spp. eggs in sheep was moderate (7.21%), but no differences were found between age groups. This result differs with those reported by Pulido-Medellin *et al*. [24], who demonstrated lower prevalence values (1.1%) in sheep. Monieziosis is present in grazing cattle and sheep, especially where infected animals contaminate pastures with the eggs of the cestode. Besides, oribatid mites (intermediate hosts) have to be also present; cysticercoids or larval stages of *Moniezia* spp. develop in them and in this way, the life cycle is completed, and the infection is maintained [15]. Climate conditions and type of grass also determine the survival of the mites. Wet soils with abundant humus and vegetation permit a better living for these intermediate hosts, in contrast with dry lands where their survival is more difficult [15]. Regarding to nematodes group, parasite genera grouped under the Strongylida order showed highest prevalence values in cattle and sheep (16.5% and 31.9%), respectively, and no differences were found between the age groups. The result agrees with those reported by Pinilla *et al*. [19] who demonstrated similar prevalence values (16.3%) in cattle from Cesar state. However, this result differs with those by Orjuela *et al*. [18] who reported higher prevalence values (70.1%) for Strongylida order in cattle from North Coast of Colombia. This result also differs with other authors, who reported 60.6% and 73% prevalence in cattle from Mexico and Peru, respectively [16,21]. In contrast, this result agrees with those reported by Pulido-Medellin *et al*. [24] who demonstrated similar prevalence values in sheep from Toca municipality, Boyaca. Nevertheless, the result differs with those reported by Herrera *et al*. [40] and Zapata *et al*. [41] who demonstrated higher prevalence values (76% and 86.6%), respectively, in sheep from Antioquia state. With respect to *Strongyloides* spp. showed low prevalence in cattle and sheep (3.8% and 3.1%), respectively, and no differences were found between age groups. The result obtained agrees with those reported by Pulido-Medellin *et al*. [24] who demonstrated similar prevalence in sheep, while the result in cattle differs with those reported by other authors, who demonstrated higher prevalence values in cattle from North Coast of Colombia and Cesar state [18,19], respectively. Probably, the weather conditions during the sampling period are not the most favorable for the transmission of GI nematodes in cattle and sheep. Quiroz *et al*. [15] showed that climate is an important factor for the presence or absence of nematodes in cattle and sheep. Although there is an adaptation to the temperature factor for the development of free life stages, the humidity is fundamental. Therefore, the regions with the highest rainfall in the tropics are the most favorable for the presentation of GI nematodes. Moreover, the size of the farms, soil and pastures type, deworming, sanitary management, and hygiene conditions in the farms should be considered as a protective factor for the transmission of the nematodes.

## Conclusion

Cattle and sheep in Colombian Northeastern Mountain were infected with helminths and coccidia. The prevalence values of GI parasites were moderate in both species warranting treatment. The presence of *F. hepatica* represents a risk factor to health public. Future studies are required to evaluate the parasitic dynamics throughout the year and the impact on animal production.

## Authors’ Contributions

NUD conceived and designed the research. AAF conducted the sample collection. JCPL and AAF processing of samples in the laboratory of parasitology. JCPL carried out the data analysis and writing of the manuscript. All the authors read and approved the submitted version of the manuscript.
